# Hub Genes and Pathways Related to Lemon (*Citrus limon*) Leaf Response to *Plenodomus tracheiphilus* Infection and Influenced by *Pseudomonas mediterranea* Biocontrol Activity

**DOI:** 10.3390/ijms25042391

**Published:** 2024-02-17

**Authors:** Angelo Sicilia, Riccardo Russo, Vittoria Catara, Angela Roberta Lo Piero

**Affiliations:** Department of Agriculture, Food and Environment, University of Catania, 95123 Catania, Italy; angelo.sicilia@unict.it (A.S.); riccardo.russo.1991@gmail.com (R.R.); vcatara@unict.it (V.C.)

**Keywords:** WGCNA, transcriptome, RNAseq, lemon, mal secco, *Plenodomus tracheiphilus*, *Pseudomonas mediterranea*, biocontrol agent

## Abstract

The lemon industry in the Mediterranean basin is strongly threatened by “mal secco” disease (MSD) caused by the fungus *Plenodomus tracheiphlilus*. Leaf pretreatments with *Pseudomonas mediterranea* 3C have been proposed as innovative tools for eco-sustainable interventions aimed at controlling the disease. In this study, by exploiting the results of previously performed RNAseq analysis, WCGNA was conducted among gene expression patterns in both inoculated (Pt) and pretreated and fungus-inoculated lemon plants (*Citrus limon* L.) (3CPt), and two indicators of fungal infection, i.e., the amount of fungus DNA measured in planta and the disease index (DI). The aims of this work were (a) to identify gene modules significantly associated with those traits, (b) to construct co-expression networks related to mal secco disease; (c) to define the effect and action mechanisms of *P. mediterranea* by comparing the networks. The results led to the identification of nine hub genes in the networks, with three of them belonging to receptor-like kinases (RLK), such as HERK1, CLAVATA1 and LRR, which play crucial roles in plant–pathogen interaction. Moreover, the comparison between networks indicated that the expression of those receptors is not induced in the presence of *P. mediterranea,* suggesting how powerful WCGNA is in discovering crucial genes that must undergo further investigation and be eventually knocked out.

## 1. Introduction

Mal secco disease (MSD) is a severe vascular disease caused by the mitosporic fungus *Plenodomus tracheiphilus*. The pathogen mainly infects lemon [*Citrus limon* (L.) Burm. F.] and other citrus species and hybrids that have citron or lemon as a parent [1,2,3,4,5,6,7]. The disease’s symptoms typically start from the leaves of the upper shoots and progressively proceed downwards, causing the desiccation of twigs, branches and of the entire plant [8]. The majority of Mediterranean citrus-growing countries, with the exception of Morocco and Portugal and the east coast of the Black Sea (Georgia), are affected by MSD [9]. Although climate change is supposed to reduce the disease spread in other sites, an easing of the disease is not foreseeable in the Mediterranean area, which will continue to be hit by the disease, provoking dramatic economic losses [10]. Recently, the use of biocontrol agents (BCA), capable of counteracting plant pathogens, seemed to be one of the most promising tools substituting the use of chemical antimicrobials in the view of greater sustainability [11,12,13,14,15]. The effectiveness of biological commercial products based on *Bacillus amyloliquefaciens* against *Plenodomus tracheiphilus* was tested and it was observed that bioformulates can significantly reduce disease incidence and symptom severity in pretreated *C. volkameriana* seedlings [16]. Similarly, the combination of the *Trichoderma asperellum* strain ICC012 with *Trichoderma gamsii* strain ICC080 (Remedier^®^) was useful to limit the disease incidence and the symptom’s seriousness compared to the untreated control on *Citrus volkameriana* plants [17]. RNA sequencing (RNAseq), with the use of next-generation sequencing techniques (NGS), has offered the opportunity to isolate genes of interest, develop functional markers, and quantify gene expression [18,19,20]. In the case of mal secco disease, it provided an unprecedented high-resolution view of the transcriptional profile of the host when infected with a pathogen [5] and of the same pathogen during in planta infection [21]. Moreover, the detailed lemon plant response to the beneficial agent *Pseudomonas mediterranea* and its possible protective effects against *P. tracheiphilus* infection have been recently investigated at the molecular level [22]. Transcriptome analysis revealed that in the asymptomatic phase of mal secco disease, the fungus induces a marked reprogramming of the plant transcriptome, whereas pretreatment with *P. mediterranea* acting as BCA limits pathogen replication, drastically slows the onset of the disease and sharply reduces lemon transcriptome modifications in terms of the number of DEGs [22]. 

Weighted correlation network analysis (WGCNA) is a systems biology method clustering genes with similar expression patterns in the same module based both on the correlation among gene’s expression obtained by NGS and on the concept of the interrelatedness of life activities in plants. Therefore, it is widely used to study the biological relationship between co-expression networks and plant traits, as well as to identify key genes highly associated with traits to be used as biomarkers [23,24,25,26,27]. Here, taking advantage of the previously performed global transcriptome analysis [22], we conducted a comprehensive WGCNA among gene expression patterns and two indicators of fungal infection such as the amount of fungus DNA measured in the leaves 7 days post inoculation (DPI) and the disease index (DI) registered at 14 DPI in both *P. tracheiphilus*-inoculated (Pt) and *P. tracheiphilus*-inoculated and *P. mediterranea* 3C pretreated lemon plants (3CPt samples). The aim of this work was to identify gene modules significantly associated with those traits and to construct co-expression networks related to mal secco disease leading to the identification of hub genes in the network that might play crucial roles in plant–pathogen interaction.

## 2. Results

### 2.1. Fungus DNA and Disease Index as Affected by P. mediterranea Treatment

The amount of fungus DNA at 7 DPI (no disease symptoms) was measured both in inoculated with *P. tracheiplilus* (Pt) samples and in *P. mediterranea* pretreated and fungus-inoculated samples (3CPt) (Table S1). Table S1 shows that the amount of fungus DNA in the Pt samples was more than four times higher than that measured in *P. mediterranea* pretreated samples (3CPt). *P. mediterranea* pretreatment limits the amount of fungus DNA inside the plant, suggesting that it acts as BCA by slowing down the fungus DNA replication. Moreover, the DI was measured after 14 DPI according to the scale detailed in the Material and Methods section. Concordantly, BCA pretreatment reduces the DI value by six times compared with Pt samples (Table S1), impacting the onset of the disease. The pattern of the aforementioned parameters, amount of fungus DNA (DNA_Pt) and disease index (DI) observed in both Pt and 3CPt samples indicates that lemon leaves displayed an obvious response to *P. mediterranea* treatment when infected by *P. tracheiphilus*.

### 2.2. WCGNA Reveals Gene Modules Associated with P. tracheiphilus DNA and Mal Secco Disease Index

**Figure 1:**
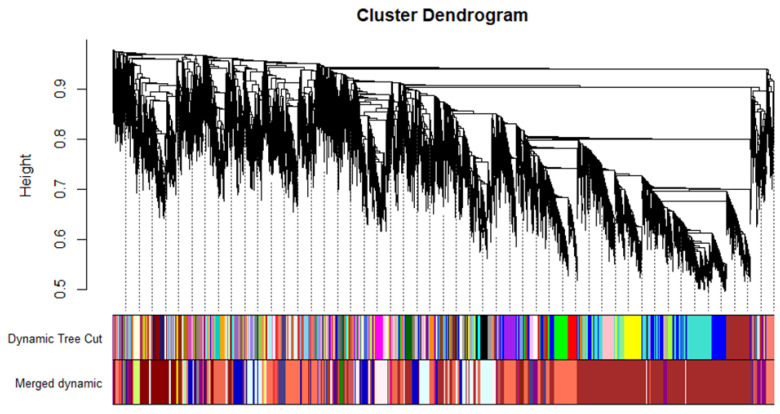
Clustering dendrogram of genes, with dissimilarity based on topological overlap, together with assigned module colors.

**Figure 2:**
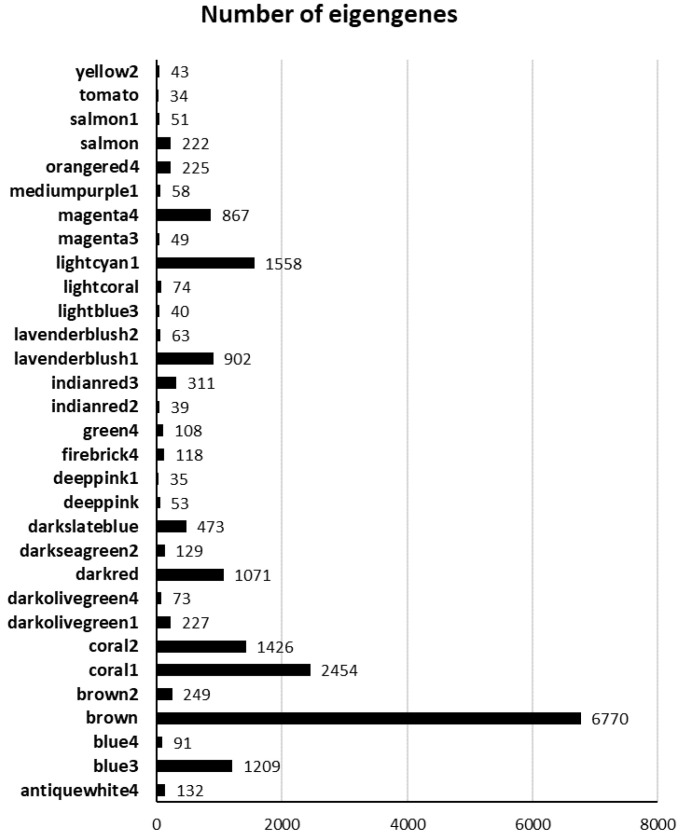
Number of eigengenes in different modules.

**Figure 3:**
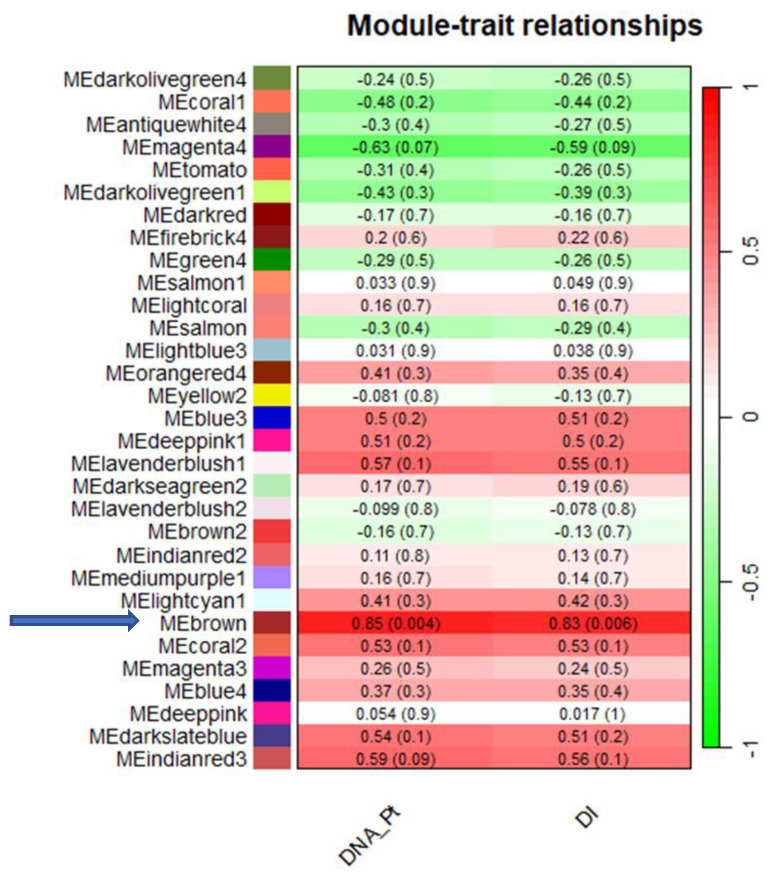
Heatmap of the correlation between modules and fungus DNA (DNA_Pt) and disease index (DI).

WGCNA was applied on the expression in lemon leaves of 19,155 unigenes (considering a FPKM > 1 as threshold) in order to correlate either fungus DNA (DNA_Pt, at 7 DPI) or DI (at 14 DPI) with gene expression patterns. Using the parameters and thresholds indicated in the Material and Methods section, these genes were grouped into 31 co-expressed modules, each corresponding to a branch of the tree (Figure 1). Figure 2 shows the number of eigengenes in each module indicating that the brown (6770), coral 1 (2454) and lightcyan 1 modules include the highest number of eigengenes (Figure 2). On the contrary, the tomato module (34) gathered the lowest number of eigengenes (Figure 2). As shown in Figure 3, illustrating the module–trait relationships, the brown module significantly correlates with both DNA_Pt and DI traits with a correlation coefficient of +0.85 and +0.83, respectively, thus indicating that the eigengenes in this module are most likely implicated both in the determination of the amount of fungus DNA and the disease index values.

**Figure 4:**
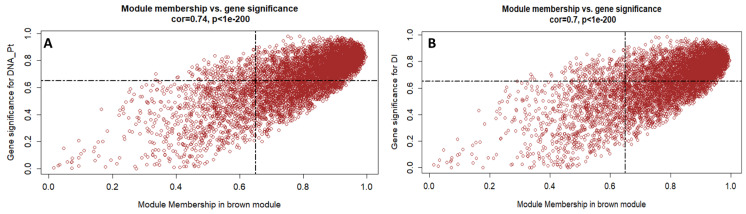
Scatterplot representing the GS and MM of the brown module. GS is plotted on the *y*-axis, MM is plotted on the *x*-axis, and each point represents an individual gene within each module. (**A**) GS for fungus DNA and (**B**) GS for DI trait.

In WGCNA, gene significance (GS) represents the correlation between a gene and a trait, and the module membership (MM) represents the correlation between an individual gene and the module eigengene. In Figure 4, the correlations between the module membership (MM) of brown modules and the gene significance (GS) with traits of interest (fungus DNA and DI) are shown. 

In particular, by fixing the MM and GS values ≥ 0.65, 3123 eigengenes were discovered to correlate with fungus DNA (DNA_Pt) and 2965 eigengenes correlate with the DI parameter (Figure S1). Interestingly, all the 2965 eigengenes correlating with the DI trait are included in the 3123 eigengenes correlating with fungus DNA, indicating that the amount of fungus DNA and the DI are strictly connected events triggering a similar plant response. Among the 3123 eigengenes, 928 were differentially expressed genes (DEGs) in the Pt vs. CK thesis, whereas only 90 of them were DEGs in the 3CPt vs. CK comparison, thus indicating (a) the wider transcriptomic response related to the amount of fungus DNA of the inoculated plants than that registered in the BCA-pretreated plant; (b) 838 DEGs are specifically upregulated in response to fungal inoculation (Pt vs. CK samples) (Figure S1). Regarding the 2965 eigengenes correlated with the DI trait, 896 DEGs were reported in the Pt vs. CK comparison and 86 were reported in the 3CPt vs. CK comparison, similarly indicating the following: (a) ampler transcriptomic reprogramming related to the disease index trait in the inoculated plants than that observed in the BCA-pretreated samples; (b) 810 DEGs are specifically upregulated in the Pt vs. CK thesis (Figure S1). Finally, Figure S1 also suggests that 32 DEGs exclusively associated with the amount of fungus DNA are upregulated in the Pt vs. CK samples (Table S2), and, among them, only 4 DEGs are also upregulated in 3CPt vs. CK samples (Table S2). In the Pt vs. CK list, snakin-2 and callose synthase 7 encoding genes are included, whereas a Brevis radix-like encoding gene is among the DEGs related to fungus DNA traits and specifically found in the 3CPt vs. CK samples (Table S2).

### 2.3. Functional Enrichment Analysis of the Eigengenes in Brown Module

**Figure 5:**
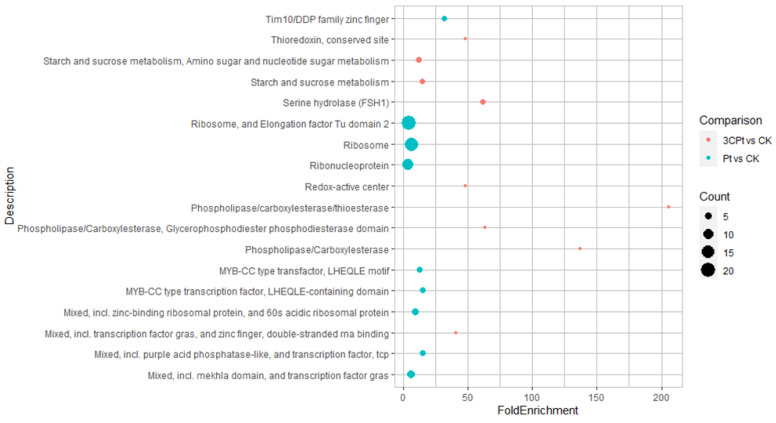
Functional enrichment of the eigengenes in the brown module. The *x*-axis represents the fold enrichment, while the *y*-axis represents the GO terms. The diameter of each circle indicates the number of genes in the category.

GO enrichment analysis of the 928 DEGs belonging to the brown module was performed as detailed in the Materials and Methods section. Figure 5 mainly shows that DEGs belonging to the Pt vs. CK comparison are particularly enriched in the “Ribosome”, “Ribonuclearprotein” and “Ribosome and Elongation factor Tu domain” categories, overall counting 51 genes related both to the fungus DNA and the DI traits (Table S3). Otherwise, “Starch and sucrose metabolism”, “Starch and sugar metabolism, amino sugar and nucleotide sugar metabolism” and “Serine hydrolase” are the main categories enriched within the 3CPt vs. CK comparison, as a whole including 16 genes related to both fungus DNA traits and to DI. Interestingly, the transcription factor (TF) category includes 11 genes related both to fungus DNA and DI traits in the Pt vs. CK comparison. Conversely, only one gene belonging to the TF category was retrieved in the 3CPt vs. CK samples, related to both fungus DNA and DI traits (Table S3). Globally, the results clearly indicate that a sharp difference occurred, not only quantitative but also qualitative, between the genes activated in response to fungal inoculation in the absence and in the presence of the BCA pretreatment (Figure 5 and Table S3). Among the 11 TFs were LOC102621228 (bHLH68-like TF) (Cluster-6461.3687), LOC102613743 (Cluster-6461.11730) and LOC102610953 (scarecrow-like protein 6) (Cluster-6461.8574), LOC102611003 (dof zinc finger protein DOF2.4) (Cluster-6461.2299), LOC102624338 (homeobox-leucine zipper protein REVOLUTA-like) (Cluster-6461.8932), LOC102628733 (TCP17 transcription factor) (Cluster-6461.3187), LOC102610499 (TCP5 transcription factor) (Cluster-6461.13409), LOC102631213 (dof zinc finger protein DOF2.5) (Cluster-6461.3056), LOC102608580 (myb family transcription factor PHL11) (Cluster-6461.5904), LOC102612487 (myb family transcription factor PHL8-like) (Cluster-6461.14903) and LOC102624965 (myb family transcription factor IPN2) (Cluster-6461.19491), suggesting that several TF families are related to both fungus DNA and DI traits in the Pt vs.CK comparison. The sole gene encoding TF included in the 3CPt vs. CK comparison was a zinc finger protein NUTCRACKER (LOC102623388) (Cluster-6461.6772) (Table S3). 

### 2.4. Functional Analysis of Co-Expression Networks

**Figure 6:**
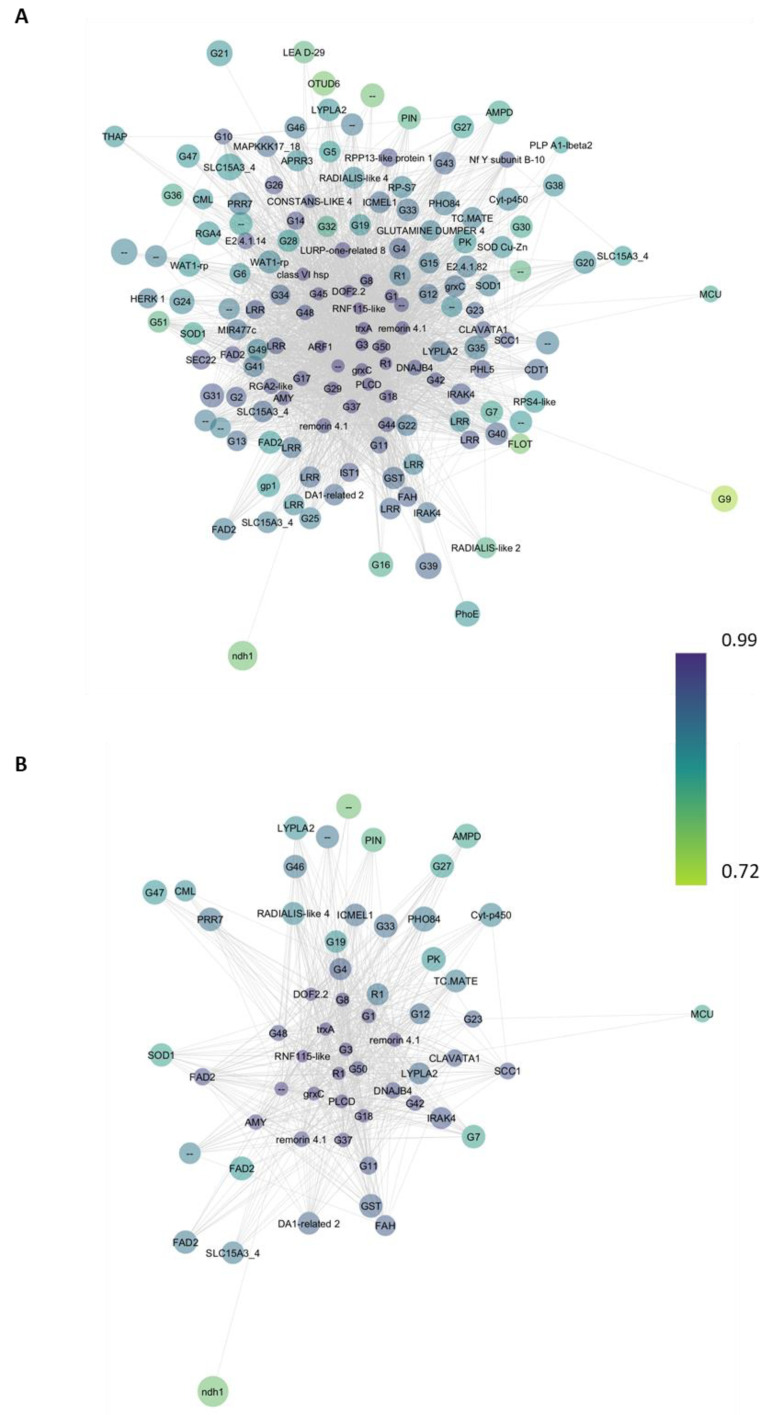
Co-expression networks of the genes related to (**A**) Pt-inoculated samples and (**B**) *P. mediterranea* pretreated samples (3CPt). The node size represents the neighborhood connectivity value, while the node color represents the module membership value.

As detailed in the Materials and Methods section, eigengenes belonging to the brown module were filtered with a weight ≥ 0.4 as the threshold and imported into Cytoscape v 3.10.1 software (Figure 6). The co-expression network of the genes related to Pt-inoculated samples (Figure 6A) shows a main large gene cluster that includes 148 genes, characterized by high connectivity (neighborhood connectivity value higher than 35), suggesting that it might represent a regulatory core network of lemon plant response to fungal infection. Among them, seven clusters encode transcription factors, belonging to the WRKY transcription factor, zinc-finger homeodomain protein 4, myb transcription factor PHL5, dof zinc finger protein DOF2.2, protein DA1-related 2 and two-component response regulator-like APRR3 families found to be involved in the plant biotic stress response. The co-expression network of the genes related to 3CPt (Figure 6B) shows a smaller unique gene cluster that includes a total of 58 genes, with neighborhood connectivity values higher than 35. Three of them belong to the protein DA1-related 2, two-component response regulator-like PRR37 and DOF1.1 transcription factor families, respectively. A complete list of Gene IDs and the corresponding network labels is in Table S5.

**Figure 7:**
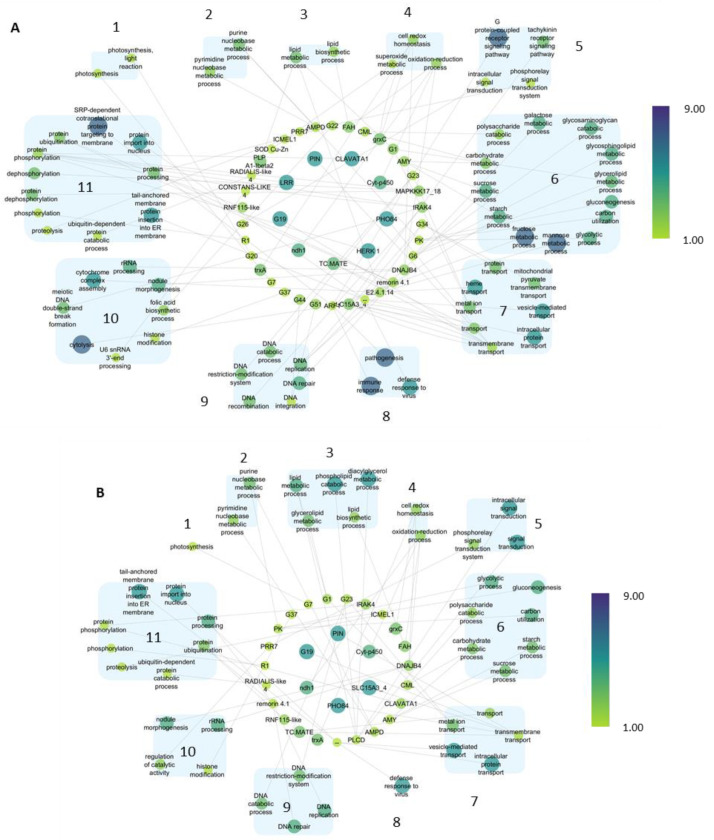
Gene networks in GO terms associated with (**A**) fungal infection (Pt, inoculated samples), (**B**) with *P. mediterranea* pretreatment (3CPt, pretreated and inoculated samples). The node size represents the neighborhood connectivity value, while the node color represents the module membership value. Numbers are the main processes in GO term. 1: photosynthesis; 2: nucleobase metabolic process; 3: lipid metabolic process; 4: redox processes; 5: signal transduction; 6: carbohydrate metabolism; 7: transport; 8: response to stimulus; 9: DNA metabolic process; 10: others; 11: protein processing.

With the aim to clarify their function, we recovered genes from the co-expression networks and grouped based on their GO classification under the following “Biological process” categories and grouped as follows: 1: photosynthesis; 2: nucleobase metabolic process; 3: lipid metabolic process; 4: redox processes; 5: signal transduction; 6: carbohydrate metabolism; 7: transport; 8: response to stimulus; 9: DNA metabolic process; 10: others; 11: protein processing. In the case of the Pt vs. CK samples, a total of 43 genes were picked from the network (Figure 7A), whereas 27 genes were recruited in the 3CPt vs. CK samples (Figure 7B) and subsequently attributed to the GO terms. As shown in Figure 7A, there were nine highly associated structural genes in the central part of the network, including PIN (Cluster-5871.3), Cyt-p450 (Cluster-6461.16030), PHO84 (Cluster-6461.10355), ndh1 (Cluster-6461.23594), LOC102626651 (Cluster-6461.3793) and three receptor-like kinases (RLK): HERK1 (Cluster-6461.1373), CLAVATA1 (Cluster-6461.9951) and LRR (Cluster-6461.18390). Due to their high neighborhood connectivity and module membership values, they could be considered candidate hub genes involved in the malsecco disease response. Similarly, Figure 7B highlights a core of five associated genes in the central part of the network PIN, Cyt-p450, PHO84, SLC15A3_4, ndh1, LOC102626651 in the 3CPt vs.CK samples. It is important to mention that receptor-like kinases (RLK) are not hub genes in the 3CPt vs. CK. Moreover, these gene networks reveal that “6: carbohydrate metabolism”, “11: protein processing” and “7: transport” categories count the highest number of subcategories in the Pt inoculated samples (Figure 7A). These subcategories are less represented in the 3CPt samples (Figure 7B), indicating that the expression of the genes involved in is not induced by BCA treatment. Interestingly, in the “8: response to stimulus” category, the pathogenesis and immune response subcategories included in the Pt vs. CK samples are no longer included in the 3CPt vs CK samples.

### 2.5. Real Time Validation of Hub Genes in Co-Expression Network

**Figure 8:**
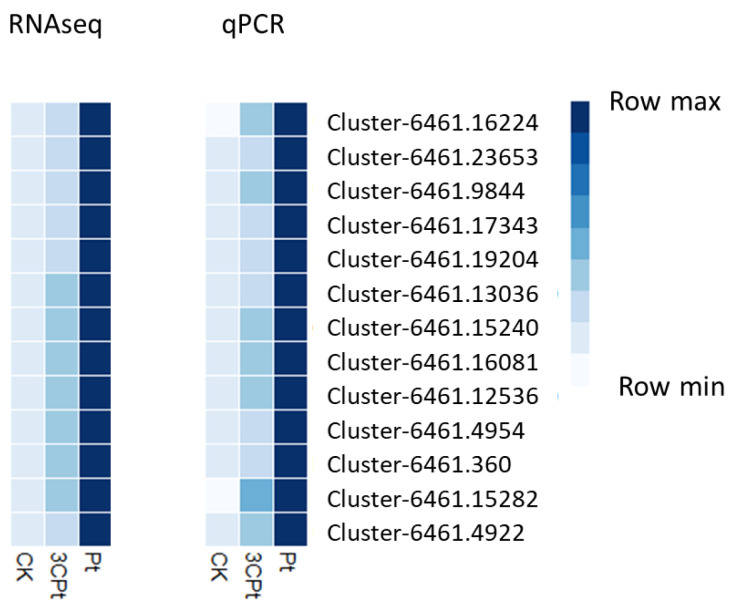
Real time RT-PCR validation of 13 hub genes. On the left, the heatmap based on RNAseq output can be observed and on the right is the heatmap constructed using real time RT PCR data (for details, see the “Section 4” section).

In order to validate the expression profile of the genes either in the Pt vs. CK or in 3CPt vs. CK samples, we selected 13 highly connected genes in the co-expression network to perform real time RT-PCR validation. As shown in Figure 8, the real time PCR results were decisively in agreement with the RNAseq results. All the genes resulted sharply up-regulated in response to both fungal inoculation (Pt) and BCA treatment (3CPt). These data also reflect the WGCNA results that indicated all the eigengenes within the brown module positively correlate with both traits (DNA_Pt) and (DI) with a correlation coefficient of +0.85 and +0.83 (see Figure 3). 

## 3. Discussion

The amount of fungus DNA measured in planta during a pathogen infection as well as the disease index evaluated in the symptomatic phase of the disease can certainly reflect both the ability of necrotrophic fungi to colonize the plant tissues and the plant potential to cope with the disease. A biological control agent (BCA) is a microorganism capable of counteracting one or more target plant pathogens by interfering with their life cycles [28,29,30]. Treatments with BCA might enhance the plant’s chances to counteract pathogens according to different mechanisms, such as the occurrence of competition for space and nutrients between the pathogen and BCA from which plants can take advantage [22]. Previously, we evaluated the effect of *P. mediterranea* pretreatment upon the transcriptome of lemon leaves inoculated with *P. tracheiplifilus* [22]. Measurements of fungus DNA and of the disease index indicated that *P. mediterranea* pretreatment sharply decreased both parameter values, therefore representing optimal markers both of mal secco disease and of the effectiveness of BCA treatment. 

In recent years, weighted gene co-expression network analysis (WGCNA) has been performed to identify hub genes in response to biotic and abiotic stress in several species, such as melon [31], sugarcane [32], maize [33] and particularly in poplar [34], where hub transcription factors and structural genes, which might play important functions in leaf blight defense, have been identified.

In this study, taking advantage of the previously performed RNAseq, we applied WGCNA to correlate gene expression levels with either fungus DNA (Pt_DNA) or disease index (DI) and to obtain the core module associated with mal secco disease. Furthermore, we used the same traits to be correlated with gene expression in samples pretreated with BCA to recruit the remaining hub genes despite the BCA pretreatment. The analysis of the module–trait relationships suggested that the brown module significantly correlates with both DNA_Pt and DI traits with very high correlation coefficients, thus indicating that the eigengenes in this module are most likely implicated in the mal secco disease response. 

The analysis of the data clearly indicated that, in the face of a discrete number of DEGs specifically found in the brown module in the Pt vs. CK samples (928), a restricted amount of DEGs were found in the 3CPt vs. CK samples (90), confirming previous data highlighting limited and lower transcriptome reprogramming in response to *P. mediterranea* pretreatment in inoculated lemon leaves [22]. Interestingly, the results indicated that 32 DEGs are specifically associated with the amount of fungus DNA in the Pt vs. CK samples. Among them, a snakin-2 encoding gene, a low molecular weight antimicrobial peptide, was retrieved. Snakins are considered as phytohormone signal transducers and integrators, closely related to the processes of biosynthesis and signal transduction of phytohormones including GA, abscisic acid (ABA) and brassinosteroids. In particular, in tomato, it prevents translocation of pathogens into plants by forming pores in the pathogen’s cell membrane [35]. Moreover, callose synthase 7 encoding gene is also on the Pt vs. CK gene list. Callose, under abiotic and biotic stress conditions, accumulates rapidly, reducing the functionality of the phloem and interfering with the transport of carbohydrates from the source organs (mainly leaves) to the sink organs (roots, flowers, fruits) [36,37] (Table S2). Regarding the DEGs related to fungus DNA traits specifically found in the 3CPt vs. CK samples, a Brevis radix-like protein, belonging to a conserved gene family differentially regulated by hormones and abiotic stresses, was recovered [38]. 

The functional enrichment analysis revealed that different categories are enriched between the Pt vs. CK and 3CPt vs. CK samples, accounting for an actual qualitative as well as quantitative difference in the transcriptomic response, especially regarding the DEG-encoding transcription factors. Many of them belong to TF families well-known to participate in plant response to biotic stress. In particular, LOC102624338 (homeobox-leucine zipper protein REVOLUTA-like) is a key regulatory node of development and plant immunity through its role in salicylic acid (SA) metabolic-pathway-mediated pathogen defense [39,40].

The gene networks in the GO term of the Pt vs CK samples showed nine highly associated structural genes, including three receptor-like kinases (RLK) such as HERK1, CLAVATA1 and LRR known to establish signaling circuits to transduce information and eventually activating processes directing growth, development, stress responses, and disease resistance [41]. To confirm their role in plant response to pathogens, it has been recently shown that the overexpression of the peanut CLAVATA1-like leucine-rich repeat receptor-like kinase confers increased resistance to bacterial wilt in tobacco [42]. It is important to mention that receptor-like kinases (RLK) are not hub genes in the 3CPt vs. CK, suggesting that the BCA treatment does not allow the plant to detect the presence of the pathogen. 

Interestingly, the “8: response to stimulus” category including pathogenesis and immune response [43] subcategories in the Pt vs. CK are no longer included in the 3CPt vs. CK samples. This result is in accordance with transcriptomic data showing that fungal infection induces the downregulation of SA and ethylene signal transduction cascades, probably blocking the activation of both Systemic Acquired Resistance (SAR) [44,45,46] and Induced Systemic Resistance (ISR) [22,47]. Conversely, genes involved in SAR or ISR were not deregulated in the samples pretreated with *P. mediterranea*, supporting the hypothesis that BCA treatment hindered the onset of the disease [22].

## 4. Materials and Methods

### 4.1. Preparation of Pseudomonas mediterranea Cell-Based Bioformulation and of Fungal Pathogen Inoculum

The bacterial inoculum was obtained from cultures of the *P. mediterranea* strain PVCT 3C as detailed in the work of Sicilia et al. [22]. Potted lemon ‘Femminello Siracusano 2Kr’ plants were treated by aerial spraying of the foliage with the bacterium suspension both at three days and one day before fungal pathogen inoculation. The inoculum of *P. tracheiphilus* (Petri) Gruyter, Aveskamp & Verkley (syn. *Phoma tracheiphila*) consisted of vial phialoconidia of the PVCT Pt57 strain obtained in carrot broth suspended in dH_2_O at a concentration of 10^6^ mL^−1^ cfu [48]. To evaluate the effect of the treatment with the biocontrol agent, the following theses were defined as follows: CK thesis, plants pretreated with water and H_2_O inoculated; Pt thesis: plants treated with water and *P. tracheiphilus* inoculated; 3CPt thesis, plants pretreated with the biocontrol agent and *P. tracheiphilus* inoculated. All the downstream analyses were performed considering either the Pt vs CK (inoculated versus control plants) or 3CPt vs CK (pretreated and inoculated versus control plants) comparisons. The leaves intended either for DNA or RNA extractions were collected at 7 DPI, which coincided with the absence of symptoms [22]. DNA extraction and real-time PCR assay to confirm fungal infection were performed with the method described by [49]. Successively, symptoms were recorded at 14 DPI according to an empirical symptom-based scale [50] slightly modified by Dimaria et al. [48], which uses the following values: 0, no sign of infection; 1, chlorotic halo around the inoculation point; 2, chlorosis of the veins near the point of inoculation; 3, nerve chlorosis extending on both sides of the inoculation point, which reaches the central vein and the margin; 4, widespread chlorosis and/or necrotic browning starting from the ribs. The disease index was then obtained by the following formula: DI = Σ(Scale value × Number of inoculated points in that rating)/Total number of inoculated of points.

### 4.2. Transcriptome and Sequencing

RNA extraction, quantification and quality control procedures are detailed in the work of Sicilia et al. [22]. The samples were sent to Novogene for high-throughput sequencing (Illumina, San Diego, CA, USA). Libraries were generated as reported in [5,18]. De novo assembly of the transcriptome was performed using Trinity (version r20140413p1), hierarchical clustering was performed with Corset (version v1.05, https://github.com/Oshlack/Corset/wiki) to remove redundancies (parameter -m 10), and the longest transcripts of each cluster were selected as unigenes [22,51]. Gene expression levels were estimated via RSEM (version v1.2.26, http://deweylab.github.io/RSEM/) [52] with bowtie2 mismatch 0 parameters to map the Corset-filtered transcriptome. Differential expression analysis was performed using the R DESeq package (version 1.12.0, padj ≤ 0.05, https://bioconductor.org/packages/release/bioc/html/DESeq.html). Genes with a *padj* value ≤ 0.05 were assigned as differentially expressed. A log_2_FoldChange threshold of 0.58 (1.5-fold change) was adopted [22]. 

### 4.3. Weighted Correlation Networks Analysis

Co-expression analysis was performed using the weighted correlation gene co-expression network analysis (WGCNA) package in R [23] in order to identify gene clusters with highly correlated expression profiles (hub genes). In detail, an adjacency matrix was created using FPKM values of all the unigenes obtained by RNAseq with a threshold of FPKM > 1. The pickSoftThreshold function was used to choose the proper soft-thresholding power [23]. In particular, for each analysis, the lowest power for which the scale-free topology fit index reaches 0.80 was used. The specific WGCNA analysis parameters in this study were set as follows: soft powers β = 6, WGCNA ‘mergeCutHeight’ = 0.25. The adjacency matrix was transformed into a topological overlap matrix (TOM) as well as the corresponding dissimilarity (1-TOM). Afterwards, a hierarchical clustering dendrogram of the 1-TOM matrix was constructed to classify the similar gene’s expression into different gene co-expression modules. To achieve high reliability of the results, the minimum number of genes was set to 30, and the sensitivity was set to 3.0. The relationships between each module and the *Plenodomus tracheiphilus* DNA content (DNA Pt) and the disease index (DI) was estimated by calculating the Pearson’s correlation using the module eigengene values. Therefore, modules with high correlation coefficients and with a correlation *padj* ≤ 0.05 were selected for subsequent analysis. Gene significance (GS) and module membership (MM) were calculated and a threshold of 0.65 was applied.

### 4.4. Functional Enrichment of Module Genes

Based on differentially expressed genes (DEGs) assigned to the brown module, the pathway’s enrichment was accomplished by using the ShinyGO V0.77 online tool [53], setting “all the available gene set” in the pathway database. Pathways with FDR ≤ 0.05 were considered significantly enriched.

### 4.5. Network Construction 

Eigengenes within the brown module were filtered with a weight ≥ 0.4 as the threshold and imported into Cytoscape v 3.10.1 software [54]. In order to achieve better network resolution, DEGs for both Pt vs CK and 3CPt vs CK comparisons were selected for visualization and a log_2_FC ≥ 1.4 threshold was applied. Three clusters were erased as they were associated with both neighborhood connectivity and degrees equal to zero. The Biological Process GO term annotation of each DEG was used for GO network construction, and biological processes were clustered based on the main processes in GO terms. 

### 4.6. Validation of Hub Genes by Quantitative Real-Time PCR Analysis 

Thirteen hub genes with high neighborhood connectivity and module membership in the co-expression networks were selected for qRT-PCR validation. The total RNA (2.5 µg) was reverse transcribed using the SuperScript Vilo cDNA synthesis kit by Thermo Fischer Scientific, Waltham, MA, USA, according to the manufacturer’s instructions. Real-time qRT-PCR was carried out with the PowerUp SYBR Green Master mix by Thermo Fischer Scientific. All the genes were normalized with *Citrus clementina* actin (LOC18039075). All reactions were performed in triplicate and fold change measurements calculated with the 2^−∆∆CT^ method. The selected DEGs and their corresponding primer sequences are provided in Table S4.

## 5. Conclusions

In this study, the application of WCGNA led to the identification of gene modules significantly correlated with two fungal infection indicators, i.e., the amount of fungus DNA measured in planta and the disease index. Moreover, co-expression networks were constructed, allowing us to retrieve hub genes and pathways related to mal secco disease. Three of them belong to the receptor-like kinases (RLK) gene family, which play pivotal roles in plant–pathogen interaction. The comparison between networks related to the Pt vs. CK and 3CPt vs. CK samples mainly indicated that the expression of those receptors is not induced in the presence of *P. mediterranea*, and that overall, the “8: response to stimulus” GO term category (immune response, pathogenesis) is only slightly represented, thus confirming that *P. mediterranea* might prevent the pathogen from being perceived.

## Data Availability

The *Citrus limon* transcriptome was submitted to NCBI (https://www.ncbi.nlm.nih.gov/geo/) under the accession number GSE227934.

## Supplementary Materials

The following supporting information can be downloaded at: https://www.mdpi.com/article/10.3390/ijms25042391/s1.

## References

[1] Nigro F., Ippolito A., Salerno M.G. (2011). Mal secco disease of citrus: A journey through a century of research. J. Plant Pathol..

[2] Migheli Q., Cacciola S.O., Balmas V., Pane A., Ezra D., Di San Lio G.M. (2009). Mal secco disease caused by *Phoma tracheiphila*: A potential threat to lemon production worldwide. Plant Dis..

[3] Catalano C., Di Guardo M., Distefano G., Caruso M., Nicolosi E., Deng Z., Gentile A., La Malfa S.G. (2021). Biotechnological Approaches for Genetic Improvement of Lemon (*Citrus limon* (L.) Burm. f.) against Mal Secco Disease. Plants.

[4] Strano C.P., Bella P., Licciardello G., Caruso A., Catara V. (2017). Role of secondary metabolites in the biocontrol activity of *Pseudomonas corrugata* and *Pseudomonas mediterranea*. Eur. J. Plant Pathol..

[5] Russo R., Sicilia A., Caruso M., Arlotta C., Di Silvestro S., Gmitter F.G., Nicolosi E., Lo Piero A.R. (2021). De Novo transcriptome sequencing of rough lemon leaves (*Citrus jambhiri* Lush.) in response to *Plenodomus tracheiphilus* infection. Int. J. Mol. Sci..

[6] Di Guardo M., Moretto M., Moser M., Bianco L., Gentile A. (2023). De novo assembly of Citrus limon and target-sequence genotyping toward the detection of genes involved in tolerance to ‘mal secco’ disease. Acta Hortic..

[7] Catalano C., Di Guardo M., Distefano G., Gentile A., La Malfa S., Kole C. (2022). Genetic Improvement of Citrus Limon (*L. Burm* f.) for Resistance to Mal Secco Disease. Genomic Designing for Biotic Stress Resistant Fruit Crops.

[8] Nigro F., Ippolito A., Salerno M.G. (2015). Searching for Citrus rootstocks resistant to mal secco disease: A review. Acta Hortic..

[9] EPPO Global Database. https://gd.eppo.int/taxon/DEUTTR.

[10] Krasnov H., Ezra D., Bahri B.A., Cacciola S.O., Meparishvili G., Migheli Q., Blanket L. (2022). Potential distribution of the citrus Mal Secco disease in the Mediterranean basin under current and future climate conditions. Plant Pathol..

[11] Conrath U., Beckers G.J.M., Langenbach C.J.G., Jaskiewicz M.R. (2015). Priming for enhanced defense. Annu. Rev. Phytopathol..

[12] Kalai-Grami L., Karkouch I., Naili O., Slimene I.B., Elkahoui S., Zekri R.B., Touati I., Mnari-Hattab M., Hajlaoui M.R., Limam F. (2016). Production and identification of iturin A lipopeptide from *Bacillus methyltrophicus* TEB1 for control of *Phoma tracheiphila*. J. Basic Microbiol..

[13] Palmieri D., Ianiri G., Del Grosso C., Barone G., De Curtis F., Castoria R., Lima G. (2022). Advances and perspectives in the use of biocontrol agents against fungal plant diseases. Horticulturae.

[14] Puglisi I., Lo Cicero L., Lo Piero A.R. (2013). The glutathione S-transferase gene superfamily: An in silico approach to study the post translational regulation. Biodegradation.

[15] Kelbessa B.G., Dubey M., Catara V., Ghadamgahi F., Ortiz R., Vetukuri R.R. (2023). Potential of plant growth-promoting rhizobacteria to improve crop productivity and adaptation to a changing climate. CABI Rev..

[16] Aiello D., Leonardi G.R., Di Pietro C., Vitale A., Polizzi G. (2022). A new strategy to improve management of Citrus Mal Secco disease using bioformulates based on *Bacillus amyloliquefaciens* strains. Plants.

[17] Leonardi G.R., Polizzi G., Vitale A., Aiello D. (2023). Efficacy of Biological Control Agents and Resistance Inducer for Control of Mal Secco Disease. Plants.

[18] Sicilia A., Testa G., Santoro D.F., Cosentino S.L., Lo Piero A.R. (2019). RNASeq analysis of giant cane reveals the leaf transcriptome dynamics under long-term salt stress. BMC Plant Biol..

[19] Sicilia A., Santoro D.F., Testa G., Cosentino S.L., Lo Piero A.R. (2020). Transcriptional response of giant reed (*Arundo donax* L.) low ecotype to long-term salt stress by unigene-based RNAseq. Phytochemistry.

[20] Kong P., Li X., Gouker F., Hong C. (2022). cDNA Transcriptome of Arabidopsis reveals various defense priming induced by a broad-spectrum biocontrol agent *Burkholderia* sp. SSG. Int. J. Mol. Sci..

[21] Sicilia A., Russo R., Caruso M., Arlotta C., Di Silvestro S., Gmitter F.G., Gentile A., Nicolosi E., Lo Piero A.R. (2022). Transcriptome analysis of *Plenodomus tracheiphilus* infecting rough lemon (*Citrus jambhiri* Lush.) indicates a multifaceted strategy during host pathogenesis. Biology.

[22] Sicilia A., Catara V., Dimaria G., Scialò E., Russo M., Gentile A., Lo Piero A.R. (2023). Transcriptome analysis of lemon leaves (*Citrus limon*) infected by *Plenodomus tracheiphilus* reveals the effectiveness of Pseudomonas mediterranea in priming the plant response to mal secco disease. J. Plant Int..

[23] Langfelder P., Horvath S. (2008). WGCNA: An R package for weighted correlation network analysis. BMC Bioinform..

[24] Ju Z., Cao D., Liang Y., Tian H., Zhu B., Luo Y. (2018). Mining Fruit Ripening-related Transcription Factors in Tomato by Weighted Gene Co-expression Network Analyses (WGCNA). J. Chin. Inst. Food Sci. Technol..

[25] Wu Q., Pan Y.-B., Su Y., Zou W., Xu F., Sun T., Grisham M.P., Yang S., Xu L., Que Y. (2022). Comprehensive and Dynamic Gene Co-Expression Network That Associates with Smut Resistance in Sugarcane. Int. J. Mol. Sci..

[26] Li Z.-X., Zhang W.-L., Jue D.-W., Liu X., Jiang Y.-S., Tang J.-M. (2022). Transcriptome Changes Induced by Botrytis cinerea Stress and Weighted Gene Co-expression Network Analysis (WGCNA) in Actinidia chinensis. Plant Mol. Biol. Rep..

[27] Azam M., Zhang S., Li J., Ahsan M., Agyenim-Boateng K.G., Qi J., Feng Y., Liu Y., Li B., Qiu L. (2023). Identification of hub genes regulating isoflavone accumulation in soybean seeds via GWAS and WGCNA approaches. Front. Plant Sci..

[28] Corke A.T.K., Baker K.F., Cook R.J. (1974). Biological Control of Plant Pathogens.

[29] Raymaekers K., Ponet L., Holtappels D., Berckmans B., Cammue B.P.A. (2020). Screening for novel biocontrol agents applicable in plant disease management—A review. Biol. Control.

[30] Lahlali R., Ezrari S., Radouane N., Kenfaoui J., Esmaeel Q., El Hamss H., Belabess Z., Barka E.A. (2022). Biological control of plant pathogens: A global perspective. Microorganisms.

[31] Shen Q., Wu X., Tao Y., Yan G., Wang X., Cao S., Wang C., He W. (2022). Mining Candidate Genes Related to Heavy Metals in Mature Melon (*Cucumis melo* L.) Peel and Pulp Using WGCNA. Genes.

[32] Tang Y., Li J., Song Q., Cheng Q., Tan Q., Zhou Q., Nong Z., Lv P. (2023). Transcriptome and WGCNA reveal hub genes in sugarcane tiller seedlings in response to drought stress. Sci. Rep..

[33] Yu T., Zhang J., Cao J., Ma X., Li W., Yang G. (2023). Hub Gene Mining and Co-Expression Network Construction of Low-Temperature Response in Maize of Seedling by WGCNA. Genes.

[34] Wang R., Wang Y., Yao W., Ge W., Jiang T., Zhou B. (2023). Transcriptome Sequencing and WGCNA Reveal Key Genes in Response to Leaf Blight in Poplar. Int. J. Mol. Sci..

[35] Iqbal A., Khan R.S. (2023). Snakins: Antimicrobial potential and prospects of genetic engineering for enhanced disease resistance in plants. Mol. Biol. Rep..

[36] Nishimura M.T. (2003). Loss of a callose synthase results in salicylic acid—Dependent disease resistance. Science.

[37] Xia Z., Ye Y., Hu C., Wang H., Zheng L., Hu Y., Sheng L., Xing J., Jia W., Wang Y. (2024). Exogenous melatonin orchestrates multiple defense responses against Botrytis cinerea in tomato leaves. Plant Stress.

[38] Tiwari S., Muthusamy S.K., Roy P., Dalal M. (2023). Genome wide analysis of BREVIS RADIX gene family from wheat (*Triticum aestivum*): A conserved gene family differentially regulated by hormones and abiotic stresses, *Int*. J. Biol. Macromol..

[39] Dempsey D.A., Vlot A.C., Wildermuth M.C., Klessig D.F. (2011). Salicylic acid biosynthesis and metabolism. Arab. Book.

[40] Bresson J., Doll J., Vasseur F., Stahl M., von Roepenack-Lahaye E., Kilian J., Stadelhofer B., Kremer J.M., Kolb D., Wenkel S. (2022). The genetic interaction of REVOLUTA and WRKY53 links plant development, senescence, and immune responses. PLoS ONE.

[41] Jose J., Ghantasala S., Roy Choudhury S. (2020). Arabidopsis Transmembrane Receptor-Like Kinases (RLKs): A Bridge between Extracellular Signal and Intracellular Regulatory Machinery. Int J Mol Sci..

[42] Zhang C., Chen H., Zhuang R.R., Chen Y.T., Deng Y., Cai T.C., Wang S.Y., Liu Q.Z., Tang R.H., Shan S.H. (2019). Overexpression of the peanut CLAVATA1-like leucine-rich repeat receptor-like kinase AhRLK1 confers increased resistance to bacterial wilt in tobacco. J. Exp. Bot..

[43] Jones J., Dangl J. (2006). The plant immune system. Nature.

[44] van Loon L.C., Bakker P.A.H.M., Pieterse C.M.J. (1998). Systemic resistance induced by rhizosphere bacteria. Annu. Rev. Phytopathol..

[45] Fu Z.Q., Dong X. (2013). Systemic acquired resistance: Turning local infection into global defense. Annu. Rev. Plant Biol..

[46] Shine M.B., Xiao X., Kachroo P., Kachroo A. (2019). Signaling mechanisms underlying systemic acquired resistance to microbial pathogens. Plant Sci..

[47] Choudhary D.K., Prakash A., Johri B.N. (2007). Induced systemic resistance (ISR) in plants: Mechanism of action. Indian J. Microbiol..

[48] Dimaria G., Mosca A., Anzalone A., Paradiso G., Nicotra D., Privitera G.F., Pulvirenti A., Catara V. (2023). Sour orange microbiome is affected by infections of *Plenodomus tracheiphilus* causal agent of Citrus mal Secco disease. Agronomy.

[49] Licciardello G., Grasso F.M., Bella P., Cirvilleri G., Grimaldi V., Catara V. (2006). Identification and detection of *Phoma tracheiphila*, causal agent of citrus mal secco disease, by realtime polymerase chain reaction. Plant Dis..

[50] Luisi N., De Cicco V., Cutuli G., Salerno M. (1979). Ricerche su un metodo di studio della patogenicità del mal secco degli agrumi. Ann. Dell’istituto Sper. Per L’agrumicoltura.

[51] Davidson N.M., Oshlack A. (2014). Corset: Enabling differential gene expression analysis for de novo assembled transcriptomes. Genome Biol..

[52] Liu Y. (2014). Bioinformatics: The Impact of Accurate Quantification on Proteomic and Genetic Analysis and Research.

[53] Ge S.X., Jung D., Yao R. (2020). ShinyGO: A graphical gene-set enrichment tool for animals and plants. Bioinformatics.

[54] Shannon P., Markiel A., Ozier O., Baliga N.S., Wang J.T., Ramage D., Amin N., Schwikowski B., Ideker T. (2003). Cytoscape: A software environment for integrated models of biomolecular interaction networks. Genome Res..

