# Supplementary material for: Hub Genes and Pathways Related to Lemon (Citrus limon) Leaf Response to Plenodomus tracheiphilus Infection and Influenced by Pseudomonas mediterranea Biocontrol Activity
Source: Int J Mol Sci. 2024 Feb 17;25(4):2391. doi: 10.3390/ijms25042391 (PMC10889467; doi:10.3390/ijms25042391)
Supplement: Supplementary file 1 [file ijms-25-02391-s001.zip › Figure S1.pdf]

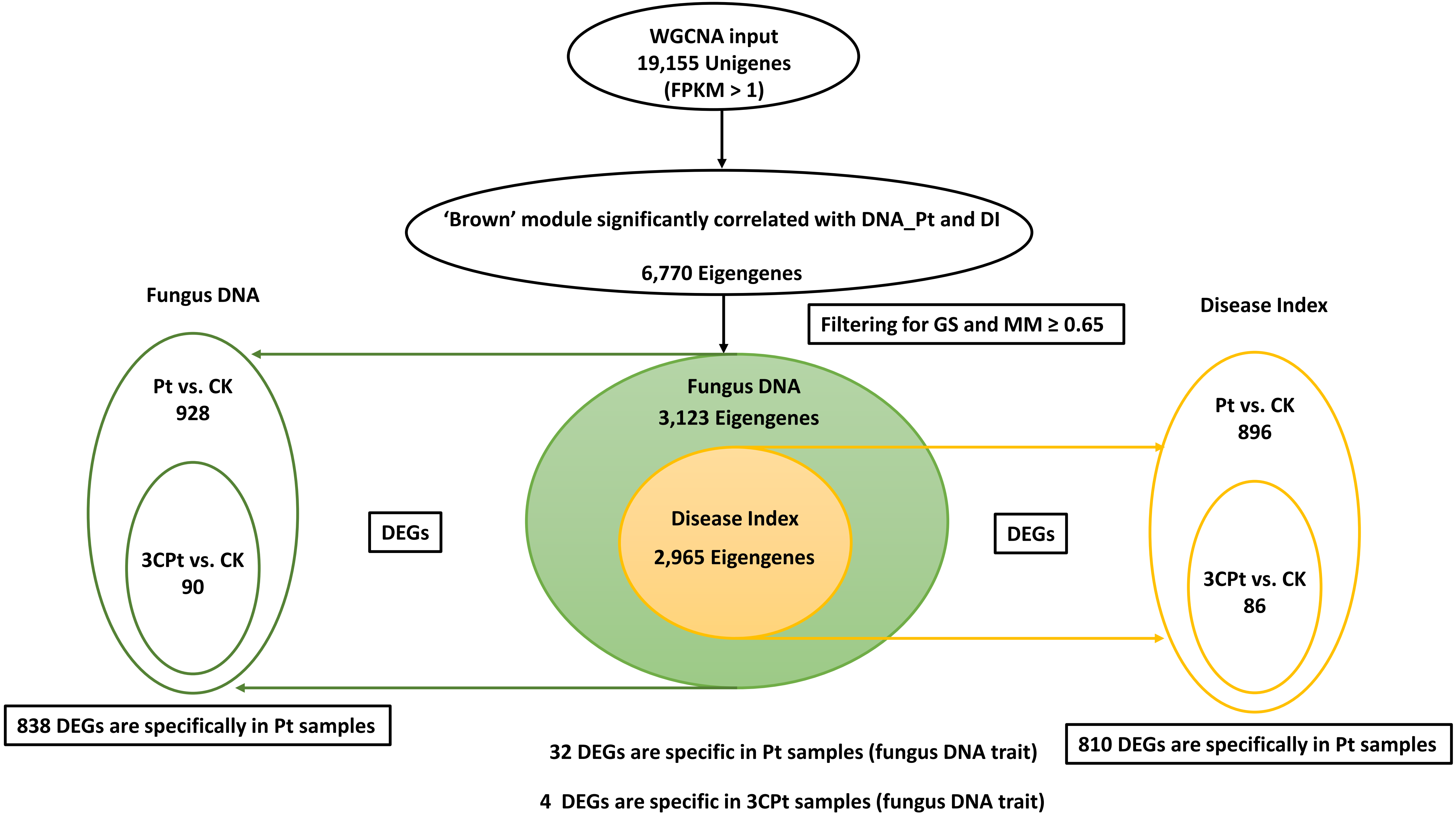
WGCNA input
19,155 Unigenes
(FPKM > 1)
'Brown' module significantly correlated with DNA_Pt and DI
6,770 Eigengenes
Fungus DNA
Filtering for GS and MM ≥ 0.65
Disease Index
Pt vs. CK
928
3CPt vs. CK
90
DEGs
Fungus DNA
3,123 Eigengenes
Disease Index
2,965 Eigengenes
DEGs
Pt vs. CK
896
3CPt vs. CK
86
838 DEGs are specifically in Pt samples
32 DEGs are specific in Pt samples (fungus DNA trait)
4 DEGs are specific in 3CPt samples (fungus DNA trait)
810 DEGs are specifically in Pt samples
