# Supplementary material for: Hub Genes and Pathways Related to Lemon (Citrus limon) Leaf Response to Plenodomus tracheiphilus Infection and Influenced by Pseudomonas mediterranea Biocontrol Activity
Source: Int J Mol Sci. 2024 Feb 17;25(4):2391. doi: 10.3390/ijms25042391 (PMC10889467; doi:10.3390/ijms25042391)
Supplement: Supplementary file 1 [file ijms-25-02391-s001.zip › Table S1.pdf]

Table S1- Values of traits considered for WGCNA analysis. Mean values of three biological replicates are reported. Different letters indicate significant difference of the trait value between samples for Student's test by p=0.05

Trait	***Pt***	***3CPt***
Fungus DNA (pg/total DNA)	17a	4b
Disease index (14 DPI)	0.18a	0.03b
