# Supplementary material for: Hub Genes and Pathways Related to Lemon (Citrus limon) Leaf Response to Plenodomus tracheiphilus Infection and Influenced by Pseudomonas mediterranea Biocontrol Activity
Source: Int J Mol Sci. 2024 Feb 17;25(4):2391. doi: 10.3390/ijms25042391 (PMC10889467; doi:10.3390/ijms25042391)
Supplement: Supplementary file 1 [file ijms-25-02391-s001.zip › Table S3.pdf]

**Enriched Pathway**	**Number of genes**			
	Pt vs. CK		3CPt vs.CK	
	Pt_DNA	DI	Pt_DNA	DI
Ribosome, Elongation factor, Ribonucleoprotein	51	51	--	--
Starch and sucrose metabolism, amino sugar and nucleotide sugar metabolism	--	--	7	7
Serine hydrolase (FSH1)	--	--	3	3
Phospholipase/carboxylesterase/thioesterase	--	--	6	6
Transcription factor	11	11	1	1

Table S3 – Number of genes included in the functional categories retrieved using ShinyGO V0.77 on line tool, related to fungus DNA (Pt_DNA) and to disease index (DI) traits.
