# Supplementary material for: Hub Genes and Pathways Related to Lemon (Citrus limon) Leaf Response to Plenodomus tracheiphilus Infection and Influenced by Pseudomonas mediterranea Biocontrol Activity
Source: Int J Mol Sci. 2024 Feb 17;25(4):2391. doi: 10.3390/ijms25042391 (PMC10889467; doi:10.3390/ijms25042391)
Supplement: Supplementary file 1 [file ijms-25-02391-s001.zip › Table S4.pdf]

Table S4- Primers used to validate the hub genes of the network analysis by real time PCR.

Eigengenes	Primer sequences
LOC102626833	For- 5'AGAACGAATGGAGGCTTGGG3'<br>Rev-5'CGAGTACACAGAGTTTTGCCTC3'
LOC102618019	For-5'ATGAGGTGCATGCTGTTGTTGAA3'<br>Rev-5'AAATGCCAGCGCCTCTTTTTC3'
LOC102630937	For-5'TGAAAGCACCATCTTCTGTTGT3'<br>Rev-5'TAAGGGGCCGATAACAAGCAT3'
Cluster-6461.4922	For-5'ATGGCTTCTGCCTACATCCG3'<br>Rev-5'TAGACGACTGTATCGCTGCTG3
E2.4.1.82	For-5'ATGTGATGGGCCTGATGGAC3'<br>Rev-5'ATGCCCCAATAACTCCAGTGT3'
SLC15A3_4	For-5'AGCAAAGCAATATGGCGCAG3'<br>Rev-5'TCCTGCGACTTGACTTCGTC3'
LRR<br>(Cluster-6461.13036)	For-5'AATTAAGCAATCTTCAACAACTGTG3'<br>Rev-5'TCATAATCTTTTGGCAAATCCCCC3'
GrxC	For-5'TGGTAGCAGAAAGGCCAGTG3'<br>Rev-5'TGCACCAGCTCCCTCTCTAT3'
CDT1	For-5'AGAAAGCCAAACAGTCGTCAC3'<br>Rev-5'TCTGAAATTTCGGGTTTGCCTTT3'
LOC102615567	For-5'TACTGGGCTTCCGGCTACTA3'<br>Rev-5'ACCATGCGTAACTAATGTAGATGT-3'
Cluster-6461.16224	For-5'TGCATTGCCAGAATACACGGT3'<br>Rev-5'TCGGCGGGAAGAGGATTGTA3'
MAPKKK17_18	For-5'TCAACCTCCAAGACTTCAGC3'<br>Rev-5'CCAACTCTCATCCCAACCCC3'
LRR<br>(Cluster-6461.12536)	For-5'TGCATGTCAAAACTCAACAGTGA3'<br>Rev-5'ATTGCCCAATCTGGTCAGGA3'
